# Supplementary figures and images for: Bacillus thuringiensis Cry14A family proteins as novel anthelmintics against gastrointestinal nematode parasites
Source: PLoS Negl Trop Dis. 2024 Oct 25;18(10):e0012611. doi: 10.1371/journal.pntd.0012611 (PMC11540219; doi:10.1371/journal.pntd.0012611)

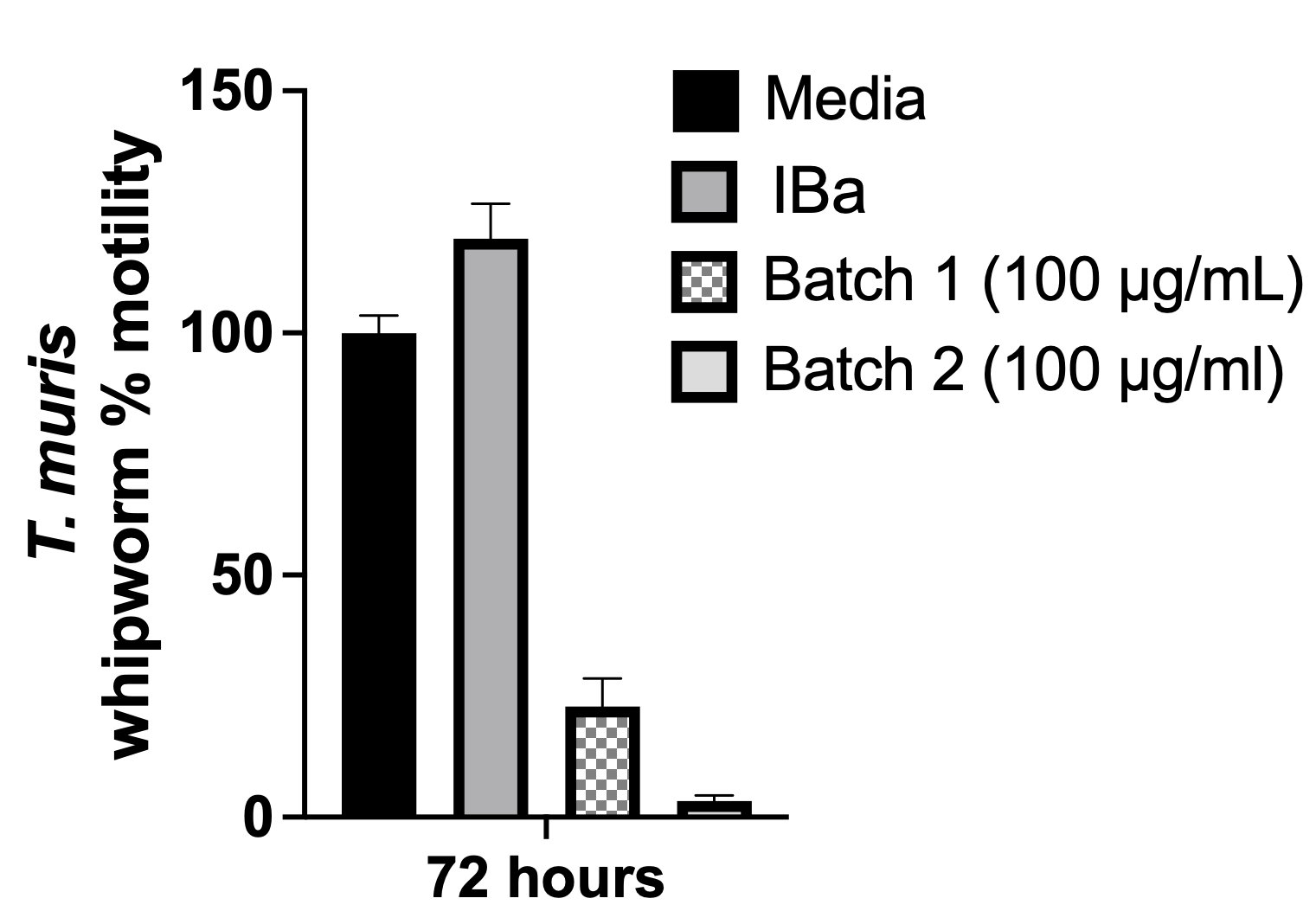

Supplement: S1 Fig — We have already demonstrated that IBa has no effect on hookworms [24] and roundworms (Fig 7B). Two independent batches of Cry14Ab IBaCC were prepared. All conditions were repeated for a total of two times and n = 12 parasites per condition. (TIF) [file pntd.0012611.s001.tif]

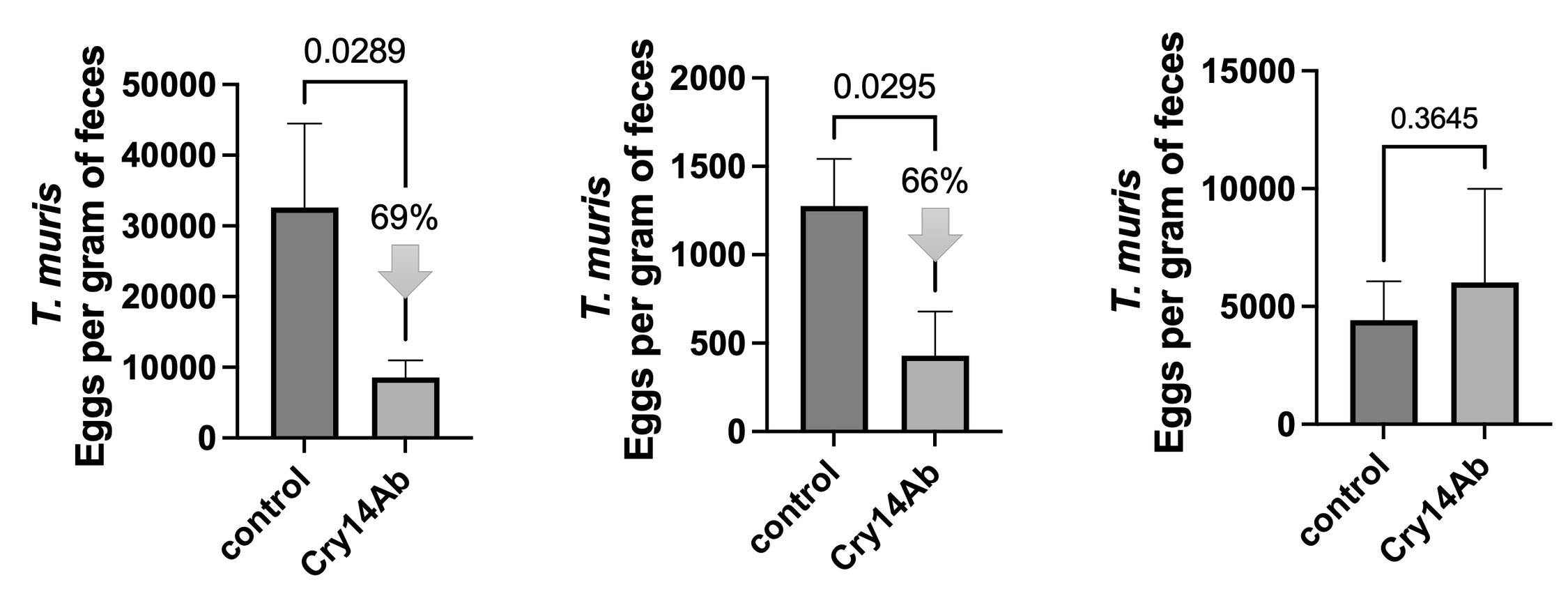

Supplement: S2 Fig — Fecal egg counts were taken seven days after direct instillation of solubilized Cry14Ab (single dose, 50 mg/kg) in 20 mM citrate buffer into the distal small intestine of the mice. P values based on one-tailed Student’s t test. Control groups received 20 mM citrate buffer (same volume). Three independent trials were carried out. In none of the experiments was a significant difference in whipworm burdens seen but in 2/3 experiments, a significant change in fecal egg counts was seen. Shown are the fecal egg counts and standard error for each group in each of the three experiments. For the experiment on the left, n = 5 control, n = 6 Cry14Ab; for the experiment in the middle, n = 4 for both groups; for the experiment on the right, n = 3 for both groups. (TIF) [file pntd.0012611.s002.tif]

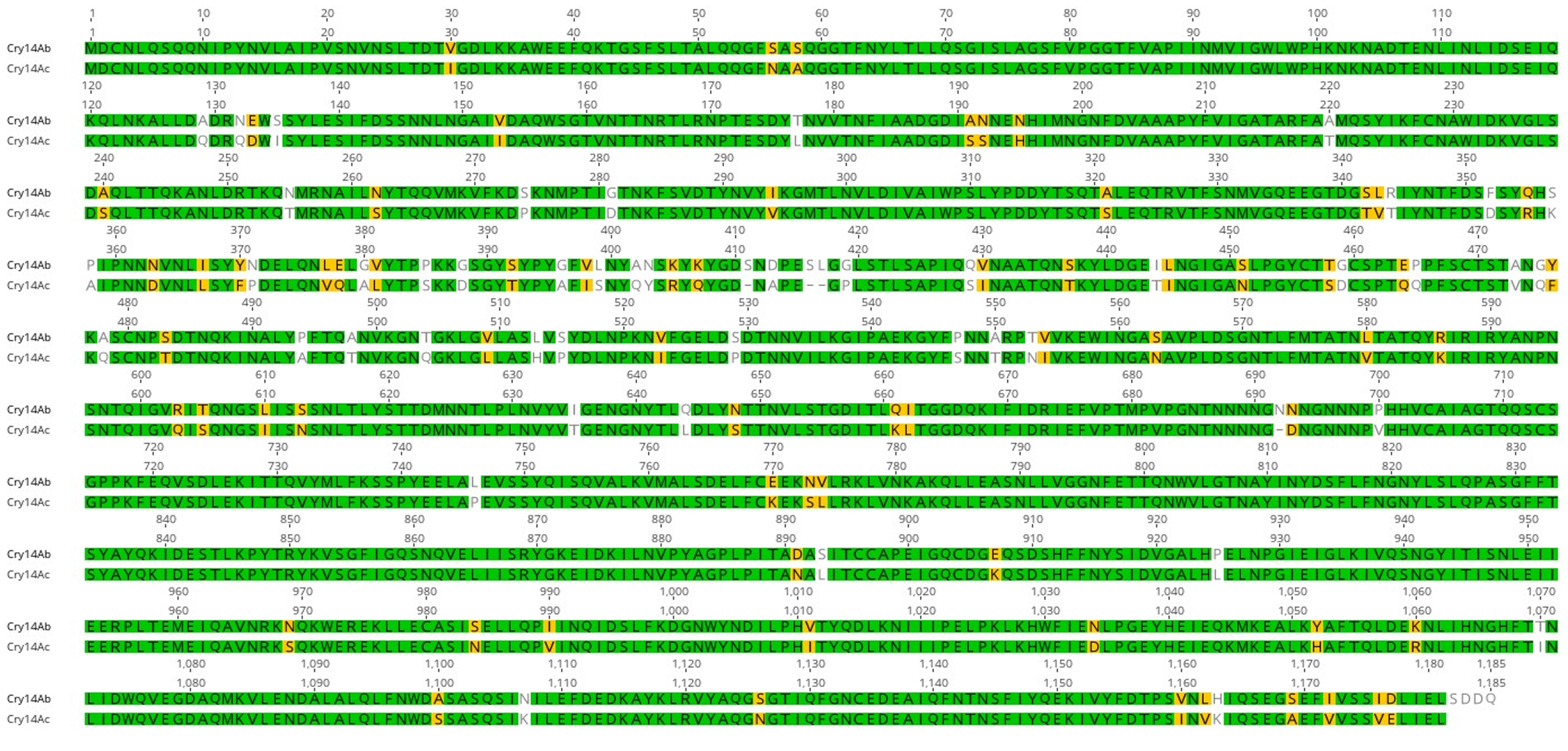

Supplement: S3 Fig — Identical residues are highlighted in green, conservative changes in yellow. Pairwise alignment was generated with Geneious Prime 2022.1.1. (TIF) [file pntd.0012611.s003.tif]
