# Supplementary material for: Bacillus thuringiensis Cry14A family proteins as novel anthelmintics against gastrointestinal nematode parasites
Source: PLoS Negl Trop Dis. 2024 Oct 25;18(10):e0012611. doi: 10.1371/journal.pntd.0012611 (PMC11540219; doi:10.1371/journal.pntd.0012611)
Supplement: Attachment — Submitted filename: Response to Reviews Cry14Ab to submit.docx [file pntd.0012611.s005.docx]

26-Aug-24

Re: PPATHOGENS-D-24-00942

Dear Drs. Diemert and Chai,

It is a great pleasure to submit our revised manuscript entitled “*Bacillus thuringiensis* Cry14A family proteins as novel anthelmintics against gastrointestinal nematode parasites.” Our responses to all comments from the reviewers are detailed individually in blue beneath each.

We look forward to further communications on this manuscript.

Thank you for your consideration.

Yours sincerely,

Raffi V. Aroian, Professor

Reviewer #1: The manuscript contains a significant number of typographical errors but since no page or line numbers are given it is too much effort for this reviewer to have to describe their location. I am happy to do this in a revised manuscript.

**Response: We have added line numbers to each page. Thank you for the suggestion.**

Reviewer #2: From the accession number given in the methods section, it appears that the Cry14Ab variant used in the study is Cry14Ab1. It would be helpful if this full designation were given at least once in the manuscript eg in the methods section, if not throughout.

**Response: We have added Cry14Ab1 the first time it is mentioned in the introduction, using Cry14Ab thereafter. The norm in the field is to omit this number.**

The work involves horse cyathosomins, which is a collection of parasites and may not be commonly known. It might be useful to help the reader by including a short sentence to introduce/describe this term.

**Response: We have added a description**.

In general, the work could be a little more clear about which controls were used in different experiments, and why.
• The use of two terms, IBa and EVC throughout the manuscript, is a little confusing. If they are the same thing, I would suggest using just one term (or explaining early that they are the same and then using just one term). If IBa is a treated form of EVC, it would be useful for this to be explained clearly at some point and with the reasons for using each as a control in particular circumstances.

**Response: We are grateful for the reviewer pointing this out. In this manuscript, we pointed out in several places they are the same thing but we agree it would be easier to simplify. We now use IBa throughout and have removed EVC.**

• Figure 2 legend, the phrasing “relative to no Cry14Ab alone” is a little confusing (if there’s no Cry14Ab, it can’t be alone). Does this mean relative to control (which control could be specified)? Figures 2 and 3 seem to be compared to a no Cry14Ab control but it is not clear whether this is water, buffer, EVA, IBa.

**Response: We have updated the legend to Figure 2 and 3 to be more clear. In all cases, control is buffer only. We have already published at IBa has no impact on larval stage parasites and hookworm adults in vitro (Li et al., 2021). In Figure 7, we show that IBa has no significant impact on Ascaris *in vitro*. In Supplemental Figure 1, we similarly show that IBa has no significant impact on whipworms.**

• Figure 4 seems to use a water control. Why isn’t the control an IBa (or extract from it)?
**Response: We have demonstrated (Li et al., 2021) that IBa has no impact on hookworm infections in this infection in hamsters.** **IBaCC is what we are advancing clinically. Clinically, the proper placebo we believe is water.**

•In the discussion it is stated that “This report is also the first instance to demonstrate that multiple members of a single family of Cry proteins are anthelmintics”. However, doi 10.1128/AEM.03505-16 indicates antihelminthic activity for Cry5B and Cry5C proteins, so the statement should be removed or modified.
**Response: Thank you. We are familiar with this research. The assays in this paper were all done against *C. elegans* or against root-knot nematode J2 larvae *in vitro*. There is no data collected from treating parasites in the host, unlike in our manuscript. Therefore, although their *in vitro* data demonstrate nematicidal activity, their data never demonstrate anthelmintic activity, which much be demonstrated in a living host (*in vivo*, which we do here). Furthermore, the stages used in the paper in question demonstrate *in vitro* activity are the J2 stage of the plant parasite; this stage is not a parasitic stage feeding on the host (it is a non-feeding infective stage).**

• Figure 5, I assume that worm burden (panels A and B) is expressed as per animal. Can this be stated (and added to the methods)? Similarly for figure 9

**Response: We have now made this clear in all *in vivo* curative figures that the numbers shown are the average worm burden or fecal egg count per rodent (hamster or mouse).**

• Figure 6, please state here or elsewhere, which variant of Cry5Ba was used.

**Response: Added in the materials and methods section.**

• Figure S3: what algorithm was used to identify and assign conserved residues? Different methods will identify different residues as conserved. I am surprised to see at residue 770 for example, the acidic E marked as conservative with the basic K (similarly at 907)

**Response: As indicated in the manuscript, the alignment was generated with Geneious Prime. Overall, we also think this alignment sufficiently communicates the high degree of identity and the low degree of divergence, at each position, across the primary sequences. We reran the analysis using NIH BLAST to align two protein sequences. The same conservative residues pointed out by the reviewer came up as conserved. The change from amino acid E (Glutamic acid) to K (Lysine) is generally considered conservative because both amino acids are charged and polar. Even though their charges are opposite, they both still interact strongly with water and other polar molecules. This means that substituting one for the other is less likely to drastically disrupt the overall structure and function of the protein compared to a change between amino acids with vastly different properties. Thus, the E to K substitution is generally considered a conservative change because the fundamental property of being charged and polar is maintained.**

Minor typographical errors:
• “The doses use in Figure 4 would, a molar level, not ...”: use -> used
**Response: Fixed.**
• “recognize nematocidal cry”: cry -> Cry

**Response: Fixed.**
• “Among these, we synthesized, expressed, and found accession no. MF893203 as highly active”. The phrasing here might be improved eg found … synthesised, expressed or change “as” to “to be” depending on the intended meaning

**Response: Reworded.**
• “5% CO2” , make subscript

**Response: Fixed.**
• Reference 9 should be corrected to show the publication date as 2021 and to add the volume number (186). References 11, 24, 35, 40, 48 – seem to lack page numbers.
• Figure 9 legend, remove italics from “infections”

**Response: Fixed.**

**Response: Fixed.**
• Figure S2 legend “was see” -> was seen

**Response: Fixed.**


Reviewer #1: The manuscript provides convincing data that Cry14Ab and Cry14Ac have activity against GIN parasites. I do though have the following comments that would improve the presentation of the publication (see also comment above about typos).


1) In the third paragraph of the introduction it says that Cry14Ab is related to Cry5Ba - the authors should specify in which way it is related.

**Response: As indicated in the original Cry14Ab paper of Kahn et al., Cry14Ab “…falls into the phylogenetic family of Cry toxins known to be active against C. elegans, including Cry5, Cry14, and Cry21.” We have added a statement to this effect.**


2) In the 2nd results section it is stated that the dose of Cry14Ab that gives complete inhibition of H. contortus coreesponds to 380 pM. We are told that in a w/w comparison this is 10x more potent than Cry5Ba so we should be told what the difference is using a molar comparison

**Response: We are grateful for this comment. We have updated the section to include this in molar. It also gave us the opportunity to correct a typographical error in the molarity of Cry14Ab in the manuscript to 38 pm (it was correctly written on the figure as 38 pm).**

3) Although it is probably obvious to those in the field it is not obvious to others why larval stages were used in some assays and adults in others. This should be explained.

**Response: For our *in vitro* assays, adult hookworms and whipworms are used but L4 staged *Ascaris* is used. We have added the following in the manuscript to explain this: “The L4 stage (instead of the adult stage) is used for *A. suum* as STAT6KO mice expel this parasite after they reach the L4 intestinal stage but before they reach the adult intestinal stage.”**

4) The section on the identification of Cry14Ac implies that the software used is somehow capable of identifying nematicidal toxin genes. It isn't - it is only capable of identifying genes likely to be related to known pesticidal protein encoding genes. The authors should therefore explain how they narrowed down their search to nematicidals (presumably by searching for genes with homology to those known to have this activity)
**Response: This is correct. We have expanded the relevant materials and methods section “The Liesegang laboratory has reported on an *in silico* approach to detecting and characterizing novel pesticidal proteins from publicly available sequence databases based on optimized profiling using hidden Markov models (HMM) methodology [40]. In the case of Cry proteins, the group is represented by several CRY-HMMs to account for the sequence diversity and modular nature of the protein class. The search was narrowed down based on HMM scores and domain signatures similar to those of known nematicidal Cry proteins according the IDOPS tool. This HMM tailored to recognize nematicidal Cry proteins was used on UniProt,…”**

5) In many places no gap if left between the text and the in-text citation number

**Response: Fixed.**


6) Species names are not italicized in the most of the references

**Response: Fixed.**

7) Use superscript in legend to Fig 1

**Response: Done.**

8) This reviewer struggled to understand the term "no Cry14Ab alone" in the legend to Fig2

**Response: Fixed. See above.**

As a general note, it would help the reviewing process if page and line numbers were marked. **Response: Done.**
