# Supplementary material for: Bacillus thuringiensis Cry14A family proteins as novel anthelmintics against gastrointestinal nematode parasites
Source: PLoS Negl Trop Dis. 2024 Oct 25;18(10):e0012611. doi: 10.1371/journal.pntd.0012611 (PMC11540219; doi:10.1371/journal.pntd.0012611)
Supplement: Attachment — Submitted filename: Response to Reviews Cry14Ab round 2.docx [file pntd.0012611.s006.docx]

1 October 2024

Re: PNTD-D-24-00785R2

Dear Drs. Diemert and Chai,

It is a great pleasure to submit our revised manuscript entitled “*Bacillus thuringiensis* Cry14A family proteins as novel anthelmintics against gastrointestinal nematode parasites.” Our responses to all comments from the reviewers (round 2) are detailed individually in blue beneath each.

We look forward to further communications on this manuscript.

Thank you for your consideration.

Yours sincerely,

Raffi V. Aroian, Professor

Reviewer #1: The authors have addressed my previous concerns. I now only have some minor comments on presentation:

1) Pg 2 Line 12 and throughout. It is no longer standard practice to italicize in vivo and in vitro

**Response: Thank you for pointing this out. We have removed the italics.**

2) Pg 6 lines 9-10. Firstly the formatting is a bit odd with the parentheses including citation 26. Secondly are the authors sure that all those toxins that have activity against nematodes cluster together in a phylogenetic tree.
**Response: We have moved “[26]” outside the parentheses. Yes, relative to insecticidal Cry proteins, Cry14A family proteins cluster with other nematode-active Cry proteins (see references cited).**

3) Pg7 Line 14 Cry3A
**Response: Corrected.**

4) Pg9 line 18 "at molar level"
**Response: Corrected.**

5) Pg10 Line 15 "for detecting...." not "to detecting"
**Response: Corrected.**

6) Pg13 Line 6 "also a highly"

**Response: Corrected.**

7) Pg13 Line 7 change to "similar to that published for Cry5Ba [20,24]."

**Response: Corrected.**

8) Pg14 Line 9 the manuscript is rather overselling the data mining aspect. The only reported outcome of the IDOPS method was a protein with >90% sequence identity to an existing protein. A simple BLAST search would have easily found that! That is not to say that IDOPS is not a useful tool but this manuscript does not provide a strong endoresment.
**Response: We note that this section was expanded in response to comments from the reviews in the previous review round. Heiko provided this response to the current comment: “The 90% sequence similarity mentioned is not the key part of the story. While BLAST can give similar results in terms of sequence identity, it does not perform as good in functional identification. The strength of using curated profile Hidden Markov Chains in the IDOPS method lies in their ability to detect regions within proteins that are more closely tied to function, based on amino acid environments and co-occurrences. This approach provides far more accurate functional predictions than a simple BLAST similarity score.”**

**That is to say, IDOPS is better at predicting function and thus allowing us to narrow in on proteins that are more likely to be functionally conserved. Indeed, in the original paper (cited in this manuscript) from the Ibarra laboratory identifying the gene from the genome of a Bt strain that encodes *cry14Ac*, the authors stated that the gene was not allowed a cry gene name at the time as there was no evidence about its toxicity. IDOPS allowed us to prioritize and focus on this protein as an anthelmintic target.**

9) Pg15 Line 15 why is there a superscripted - in the 4D8 spo0A deletion but not in the parent strain? Does this represent the Cry- phenotype?

**Response: “-“ removed.**

10) Pg15 Line 23 the gene not the protein was synthesized (lower case italicized).

11) Pg16 Line 4 - see 9) above - yet another version
**Response: We have revised “DNA encoding…” And below, changed Cry14Ab and Cry14Ac to lower case italics.**

12) Pg17 Line 13 For the experiment

**Response: Corrected.**

13) Pg18 Line 12 space after the 2nd 20

**Response: Corrected.**

14) Pg 20 Line 20 studies was used as intact

**Response: Corrected.**

15) Pg 22 Line 2 change to "of pH3 solubilized Cry14Ab

**Response: Corrected.**

16) Legend to Fig 1 yet another way of formating the strain name (see 9) and 11) above)

**Response: We appreciate the reviewer pointing out the inconsistencies in the nomenclature . We have amended the figure legend to be consistent (“4D8 ∆*spo0A”*) throughout.**
